# Supplementary material for: Impact of a Live Attenuated Classical Swine Fever Virus Introduced to Jeju Island, a CSF-Free Area
Source: Pathogens. 2019 Nov 20;8(4):251. doi: 10.3390/pathogens8040251 (PMC6963429; doi:10.3390/pathogens8040251)
Supplement: Supplementary file 1 [file pathogens-08-00251-s001.zip › Supplemental tables (20191112)/Supplemental Table 1.pdf]

pathogens

MDPI

Supplemental Table 1. Amino acid differences between commercial LOM vaccine strains and Jeju LOM strains (2004–2007).

Vaccine	Year	Strain (Accession no)	Amino acid position												
			Erns		E1		E2		NS3	NS4B				NS5A	NS5B
			386	480	577	584	1053	1065	1165	1976	2352	2378	2383	2816	3312
Commercial LOM vaccine strains	1987	LOM-850	D	R	V	E	I	L	K	I	A	M	V	N	H
	2002	LOM (EU789580)	-	-	-	-	-	-	-	-	-	-	-	-	-
	2016	16LOM-GC00	-	-	-	V	-	-	-	-	-	-	-	-	-
		16LOM-JY00	-	-	-	-	-	-	-	-	-	-	-	-	-
		16LOM-KM00	-	-	-	-	-	-	-	-	-	-	-	-	-
		16LOM-KR00	-	-	-		-	-	-	-	-	-	-	-	-
Jeju LOM strains	2004	JJ04LOM-Tamra01	-	-	A	V	-	-	-	-	-	L	A	-	-
	2005	JJ05LOM-KSM01	N	G	M	V	-	S	R	-	V	L	A	T	-
	2006	JJ06LOM-JSY01	N	G	M	V	M	S	R	V	V	L	A	T	Y
	2007	JJ07LOM-JSM01	N	G	M	V	M	S	R	V	V	L	A	T	Y
		JJ07LOM-JSG02	N	G	M	V	M	S	R	V	V	L	A	T	Y

LOM-850: live attenuated CSF vaccine, original master seed distributed from APQA to animal medical veterinary companies (AMVC) in South Korea in 1987; LOM (EU789580); several hundred passages of the LOM850 strain in PK-15 cells. 16LOM-GC00, 16LOM-JY00, 16LOM-KM00, and 16LOM-KR00: commercial CSF vaccines from four AMVCs collected from the market in 2016.
