# Supplementary material for: Impact of a Live Attenuated Classical Swine Fever Virus Introduced to Jeju Island, a CSF-Free Area
Source: Pathogens. 2019 Nov 20;8(4):251. doi: 10.3390/pathogens8040251 (PMC6963429; doi:10.3390/pathogens8040251)
Supplement: Supplementary file 1 [file pathogens-08-00251-s001.zip › Supplemental tables (20191112)/Supplemental table 2.pdf]

Supplemental table 2. Amino acid differences between commercial LOM vaccine strains and Jeju LOM strains (2014–2018).

Vaccine	Year	Strain (Accession no)	Amino acid position														
			Npro		Erns		E1	NS3			NS4B				NS5A	NS5B	
			57	143	351	476	651	1381	1584	2006	2348	2371	2398	2483	2978	3409	3786
Commercial LOM vaccine strains	1987	LOM-850	K	L	Y	R	I	V	H	K	M	T	I	V	A	N	S
	2002	LOM (EU789580)	-	-	-	-	-	-	-	-	-	-	-	-	-	-	-
	2016	16LOM-GC00	-	-	-	-	-	-	-	-	-	-	-	-	-	-	-
		16LOM-JY00	-	-	-	-	-	-	-	-	-	-	-	-	-	-	-
		16LOM-KM00	-	-	-	-	-	-	-	-	-	-	-	-	-	-	-
		16LOM-KR00	-	-	-	-	-	-	-	-	-	-	-	-	-	-	-
Jeju LOM strains	2014	JJ14LOM-WSH01	-	-	-	-	T	-	N	-	-	-	-	A	-	-	-
	2016	JJ16LOM-YYM02	-	-	-	-	-	-	-	-	-	-	-	A	-	-	-
		JJ16LOM-WSH03	R	Q	H	S	T	I	N	I	I	I	M	A	T	S	N
		JJ16LOM-YJK08	R	Q	H	S	T	I	N	I	I	I	M	A	T	S	N
	2017	JJ17LOM-PYS03	R	Q	H	S	T	I	N	I	I	I	M	A	T	S	N
		JJ17LOM-KJS09	R	Q	H	S	T	I	N	I	I	I	M	A	T	S	N
		JJ17LOM-CCJ04	R	Q	H	S	T	I	N	I	I	I	M	A	T	S	N
		JJ17LOM-SJM06	R	Q	H	S	T	I	N	I	I	I	M	A	T	S	N
		JJ17LOM-IGS07	R	Q	H	S	T	I	N	I	I	I	M	A	T	S	N
		JJ17LOM-IGS08	R	Q	H	S	T	I	N	I	I	I	M	A	T	S	N
		JJ17LOM-LHH10	-	-	-	-	-	-	-	-	-	-	-	-	-	-	-
		JJ17LOM-JSJ12	R	Q	H	S	T	I	N	I	I	I	M	A	T	S	N
		JJ17LOM-HSJ13	R	Q	H	S	T	I	N	I	I	I	M	A	T	S	N
	2018	JJ18LOM-KGS01	R	Q	H	S	T	I	N	I	I	I	M	A	T	S	N
		JJ18LOM-KYN02	R	Q	H	S	T	I	N	I	I	I	M	A	T	S	N
		JJ18LOM-PIC03	R	Q	-	-	-	-	-	-	I	I	-	A	-	-	-

LOM-850: live attenuated CSF vaccine, original master seed distributed from APQA to animal medical veterinary companies (AMVC) in South Korea in 1987; LOM (EU789580): several hundred passages of the LOM850 strain in PK-15 cells. 16LOM-GC00, 16LOM-JY00, 16LOM-KM00, and 16LOM-KR00: commercial CSF vaccines of from four AMVCs collected from the market in 2016.
