# Supplementary material for: Impact of a Live Attenuated Classical Swine Fever Virus Introduced to Jeju Island, a CSF-Free Area
Source: Pathogens. 2019 Nov 20;8(4):251. doi: 10.3390/pathogens8040251 (PMC6963429; doi:10.3390/pathogens8040251)
Supplement: Supplementary file 1 [file pathogens-08-00251-s001.zip › Supplemental tables (20191112)/Supplemental table 3.pdf]

pathogens

MDPI

Supplemental table 3. Positively selected sites and omega values for the Jeju LOM strains.

Group	Strains	Homology nt/aa (%)	Omega value	Positively selected sites (aa position) (*P>95%; **P>99%)	
				NEB[a]	BEB[b]
Jeju LOM strains (2004–2007)	JJ04LOM-Tamra01<br>JJ05LOM-KSM01<br>JJ06LOM-JSY01<br>JJ07LOM-JSM01<br>JJ07LOM-JSG02	98.8–99.2/ 99.2–99.4	0.14088	-	237, 259, 577, 2464
Jeju LOM strains (2014–2018)	JJ14LOM-WSH01<br>JJ16LOM-YYM02<br>JJ16LOM-WSH03<br>JJ16LOM-YJK08<br>JJ17LOM-PYS03<br>JJ17LOM-KJS09<br>JJ17LOM-CCJ04<br>JJ17LOM-SJM06<br>JJ17LOM-IGS07<br>JJ17LOM-IGS08<br>JJ17LOM-LHH10<br>JJ17LOM-JSJ12<br>JJ17LOM-HSJ13<br>JJ18LOM-KGS01<br>JJ18LOM-KYN02<br>JJ18LOM-PIC03	98.2–99.4 /99.1–99.6	0.16196	**564	173, 176, 386, 564, 1337, 2676, 2988, 3605

[a] Naive Empirical Bayes (NEB) analysis; [b] Bayes Empirical Bayes (BEB) analysis.
